# Anti-β_2_-microglobulin monoclonal antibodies overcome bortezomib resistance in multiple myeloma by inhibiting autophagy

**DOI:** 10.18632/oncotarget.3251

**Published:** 2015-03-31

**Authors:** Mingjun Zhang, Jin He, Zhiqiang Liu, Yong Lu, Yuhuan Zheng, Haiyan Li, Jingda Xu, Huan Liu, Jianfei Qian, Robert Z. Orlowski, Larry W. Kwak, Qing Yi, Jing Yang

**Affiliations:** ^1^ Department of Lymphoma/Myeloma, Division of Cancer Medicine, The University of Texas MD Anderson Cancer Center, Houston, Texas, USA; ^2^ Department of Cancer Biology, Lerner Research Institute, Cleveland Clinic, Cleveland, Ohio, USA; ^3^ Cancer Research Institute and Cancer Hospital, Guangzhou Medical University, Guangzhou, China

**Keywords:** multiple myeloma, anti-β_2_M monoclonal antibody, bortezomib, autophagy, NF-κ p65

## Abstract

Our previous studies showed that anti-β_2_M monoclonal antibodies (mAbs) have strong and direct apoptotic effects on multiple myeloma (MM) cells, suggesting that anti-β_2_M mAbs might be developed as a novel therapeutic agent. In this study, we investigated the anti-MM effects of combination treatment with anti-β_2_M mAbs and bortezomib (BTZ). Our results showed that anti-β_2_M mAbs enhanced BTZ-induced apoptosis of MM cell lines and primary MM cells. Combination treatment could also induce apoptosis of BTZ-resistant MM cells, and the enhanced effect depended on the surface expression of β_2_M on MM cells. BTZ up-regulated the expression of autophagy proteins, whereas combination with anti-β_2_M mAbs inhibited autophagy. Sequence analysis of the promoter region of *beclin 1* identified 3 putative NF-κB-binding sites from –615 to –789 bp. BTZ treatment increased, whereas combination with anti-β_2_M mAbs reduced, NF-κB transcription activities in MM cells, and combination treatment inhibited NF-κB p65 binding to the *beclin 1* promoter. Furthermore, anti-β_2_M mAbs and BTZ combination treatment had anti-MM activities in an established MM mouse model. Thus, our studies provide new insight and support for the clinical development of an anti-β_2_M mAb and BTZ combination treatment to overcome BTZ drug resistance and improve MM patient survival.

## INTRODUCTION

Multiple myeloma (MM) is a clonal plasma cell neoplasm that utilizes the bone marrow (BM) microenvironment for survival and proliferation [1–3]. Current MM therapies are rarely curative, and relapse is common. Such failure implies that therapy-resistant, MM-initiating cells exist and that new therapeutics must be developed to target and eradicate these chemoresistant MM cells.

Bortezomib (BTZ) is a proteasome inhibitor used worldwide to treat MM and mantle cell lymphoma [4]. However, adverse effects and drug resistance are emerging as great challenges for its extended application [5]. Cell death and survival are regulated by the crosstalk between apoptosis and autophagy [6], and autophagy activation inhibits apoptosis through reducing caspase cleavage [7, 8]. Recent studies have shown that autophagy activation plays a role in chemotherapy drug resistance in patients with cancer [9]. In particular, BTZ treatment activates autophagy in tumor cells [10, 11]. BTZ-induced autophagy is important in BTZ drug resistance in breast cancer, suggesting that inhibiting autophagy may overcome BTZ-induced drug resistance [9].

Targeted immunotherapy with monoclonal antibodies (mAbs) is an effective and safe cancer treatment. Recent efforts have identified potential therapeutic mAbs by defining alternative or novel MM target antigens, i.e., CD40 [12, 13], interleukin-6 receptor [14], HM1.24 [15, 16], CD74 [17], CD47 [18], TRAIL-R1 [19], CS1 [20], PD-1 [21], as well as by conjugating mAbs with classic or novel drugs to specifically kill MM cells, i.e., CD56-maytansinoid (DM1) [22], CD138-DM1/DM4 [23]. Because most of these antibodies have little activity clinically in myeloma, the development of mAbs with improved cytotoxicity, targeting new and known MM-associated antigens, continues to be an active research area.

β_2_-microglobulin (β_2_M) is a part of the major histocompatibility complex (MHC) class I molecule [24]. We recently demonstrated that human β_2_M is a potential target for MM treatment [25, 26]. Our previous studies showed that anti-β_2_M mAbs have strong and direct apoptotic effects on MM and other hematological malignancies, with less toxicity to normal tissues and cells [25, 27], suggesting that anti-β_2_M mAbs might be a novel therapeutic agent for MM. Furthermore, others have reported similar results using an anti-MHC class-1 single-chain Fv diabody or anti-β_2_M antibodies to induce apoptosis in human MM [28] and other cancers [29, 30].

Here, we examined the anti-MM effects of combination treatment with anti-β_2_M mAbs and BTZ. Combination treatment inhibited BTZ-induced autophagy and increased MM cell apoptosis to overcome BTZ resistance. These results support the clinical development of anti-β_2_M mAb and BTZ combination treatment to improve MM patient outcomes.

## RESULTS

### Anti-β_2_M mAbs enhance the effects of BTZ on MM cell apoptosis

**Figure 1:**
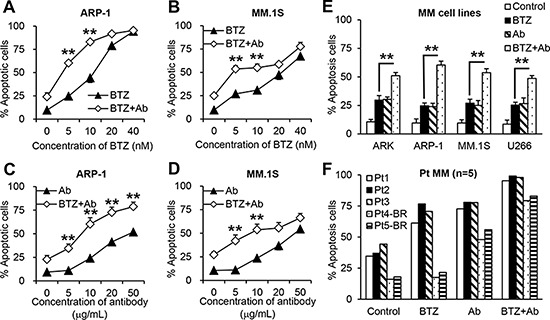
Anti-β_2_M mAbs and BTZ combination treatment in MM cells 10 μg/mL anti-β_2_M mAbs were combined with various concentrations of BTZ in ARP-1. **(A)** and MM.1S **(B)** cells. 5 nM BTZ combined with various concentrations of anti-β_2_M mAbs in ARP-1 **(C)** and MM.1S **(D)** cells. **(E)** Anti-β_2_M mAbs combined with BTZ in different MM cell lines. **(F)** Anti-β_2_M mAbs combined with BTZ in CD138^+^ patient (Pt) MM cells isolated from three BTZ-sensitive MM patients and two BTZ-resistant (BR) MM patients. After 24 hours of treatment, cell apoptosis was monitored by annexin-V binding assay. In E and F, 5 nM BTZ and 10 μg/mL anti-β_2_M mAbs was used. Summarized data from three independent experiments are shown. **P < 0.01.

To investigate the combination effects of anti-β_2_M mAbs and BTZ, MM cells were cultured in medium with different concentrations of BTZ (0 nM to 40 nM) alone or in combination with anti-β_2_M mAbs (10 μg/mL) for 24 hours. Annexin-V binding assay showed that BTZ at lower concentrations (5 nM and 10 nM) in combination with the mAbs significantly enhanced apoptosis of ARP-1 (Figure 1A) and MM.1S (Figure 1B) cells. Treatment with high concentrations of BTZ (20 nM and 40 nM) alone had strong anti-MM effects, but combination with the mAbs had no synergistic effects (Figure 1A and 1B; *P* < 0.01). Next, MM cells were cultured with various anti-β_2_M mAb concentrations (0 μg/mL to 50 μg/mL), either alone or in combination with a low (5 nM) BTZ concentration for 24 hours. Combination treatment significantly enhanced apoptosis of ARP-1 (Figure 1C) and MM.1S (Figure 1D) cells in an anti-β_2_M mAb dose-dependent manner (*P* < 0.01, compared with mAb treatment alone). Combination of anti-β_2_M mAbs (10 μg/mL) and BTZ (5 nM) was further evaluated in the MM cell lines ARK, ARP-1, MM.1S, and U266 in a 24-hour treatment. Compared to BTZ alone, combination treatment induced enhanced apoptosis by 1.5-fold in all examined MM cell lines (Figure 1E; *P* < 0.01). In line with these results, after 24-hour treatment, purified primary CD138^+^ MM cells isolated from 3 patients with MM were more sensitive to the combination treatment than BTZ treatment alone. Two other patients with relapse who had received BTZ were considered as BTZ-resistant. In these MM patient cells, BTZ treatment alone was ineffective whereas combination with anti-β_2_M mAbs increased apoptosis (Figure 1F, patients 4 and 5). Taken together, these results demonstrate that anti-β_2_M mAbs combined with BTZ is more effective against MM cells than BTZ treatment alone.

The Chou-Talalay combination index (CI) offers quantitative definitions for additive effect (CI = 1), synergism (CI < 1), and antagonism (CI > 1) in drug combinations. We applied the CI-isobol equation to study drug interactions between BTZ and anti-β_2_M mAbs. As shown in Supplementary Figure S1, combining BTZ and anti-β_2_M mAb has a synergistic effect (CI < 1) at a low concentration (fraction affected (*fa*) < 0.45). Therefore, we used low concentrations of BTZ (5 nM) and anti-β_2_M mAbs (10 μg/mL) in the following experiments.

### The combination of anti-β_2_M mAbs and BTZ overcomes BTZ resistance

**Figure 2:**
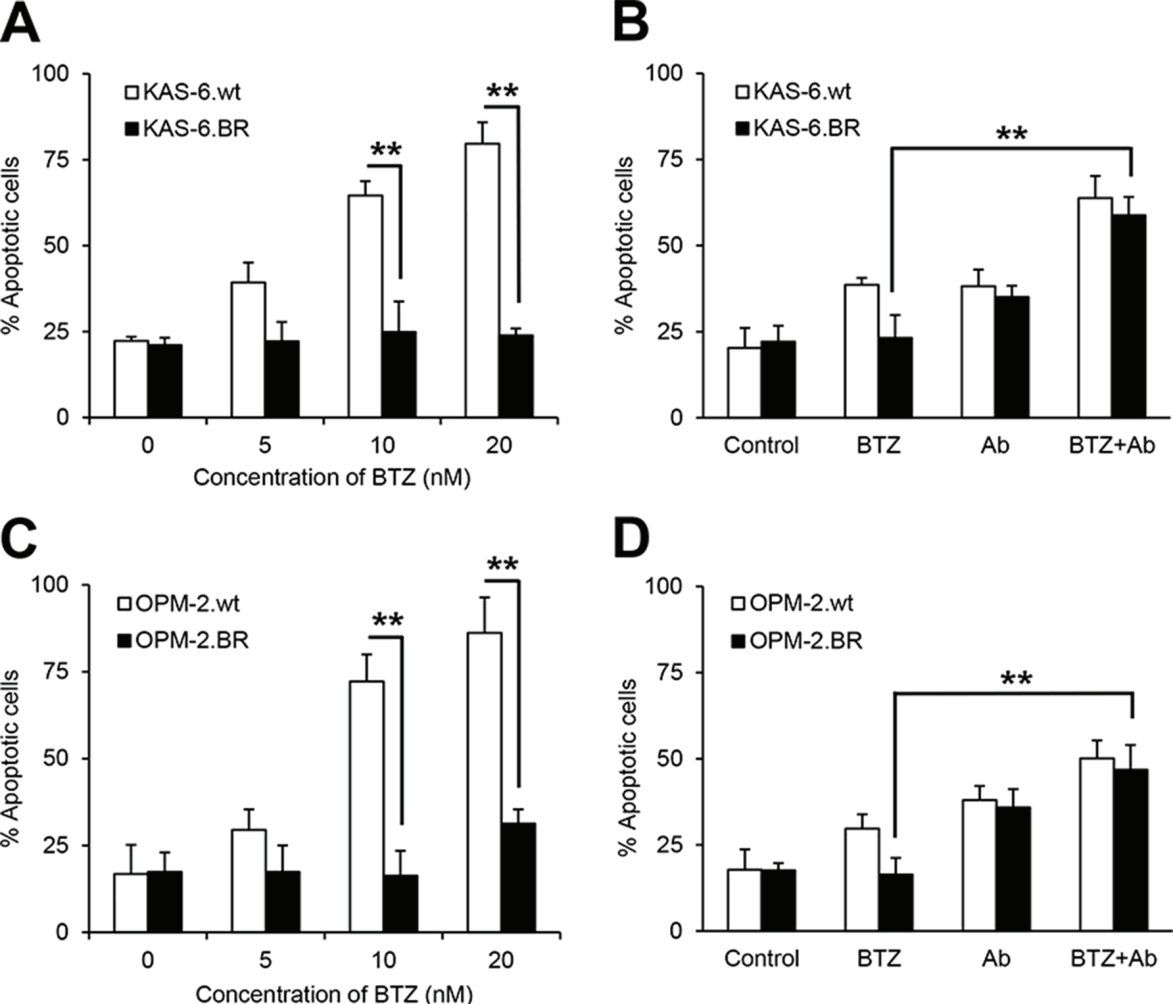
Combination of anti-β_2_M mAbs and BTZ restores the sensitivity of BTZ-resistant MM cells to BTZ treatment Wild type (wt) or BTZ-resistant (BR) KAS-6 **(A** and **B)** and OPM-2 **(C** and **D)** cells were cultured in medium with the addition of BTZ or anti-β_2_M mAbs, singly or in combination, for 24 hours. MM cell apoptosis was monitored by annexin-V binding assay. The percentage of cells undergoing apoptosis increased in a dose-dependent manner in the BTZ-sensitive cells, with no change in the percentage undergoing apoptosis in BTZ-resistant KAS-6 cells **(A)** and OPM-2 cells **(C)**, treated with various BTZ concentrations. Also shown is the increase in the percentage of cells undergoing apoptosis in either wild type or BTZ-resistant KAS-6 **(B)** and OPM-2 **(D)** cells, treated with the combination of BTZ (5 nM) and anti-β_2_M mAbs (10 μg/mL), compared with cells treated with BTZ only. Summarized data from three independent experiments are shown. **P < 0.01.

To investigate whether combining anti-β_2_M mAbs and BTZ enhances the anti-MM effects of BTZ in BTZ-resistant MM cells, we used BTZ-sensitive (KAS-6.wt and OPM-2.wt) and BTZ-resistant (KAS-6.BR and OPM-2.BR) MM cells [31]. First, we confirmed cell sensitivity to BTZ treatment, observing that BTZ treatment induced apoptosis of BTZ-sensitive cells in a dose-dependent manner, but did not induce apoptosis of BTZ-resistant cells (Figure 2A and 2C; *P* < 0.01). Next, we analyzed apoptosis of BTZ-sensitive and BTZ-resistant MM cells treated with BTZ or anti-β_2_M mAbs, alone or in combination. After 24-hour treatment, BTZ was effective in BTZ-sensitive cells but not in BTZ-resistant cells, whereas combining BTZ with anti-β_2_M mAbs induced apoptosis in both BTZ-sensitive and BTZ-resistant cells, and was more efficacious than BTZ treatment alone (Figure 2B and 2D; *P* < 0.01). These results indicate that combining anti-β_2_M mAbs with BTZ overcomes BTZ resistance in MM.

### Effects of combination treatment depends on MM cell β_2_M expression

To evaluate the significance of MM cell β_2_M expression in anti-β_2_M mAb and BTZ combination treatment-induced MM apoptosis, we used β_2_M short-hairpin RNA (shRNA)-lentiviral or β_2_M open reading frame (ORF)-lentiviral systems to knockdown or overexpress β_2_M, respectively, in MM cells. β_2_M expression was evaluated by Western blotting, quantitative real-time polymerase chain reaction (qPCR), enzyme-linked immunosorbent assay (ELISA), and flow cytometry. Significant reductions or increases in β_2_M protein (Supplementary Figure S2A and S2B) and mRNA (Supplementary Figure S2C and S2D) were observed in β_2_M shRNA- or β_2_M ORF-expressing ARP-1 and MM.1S cells compared with non-specific shRNA or control vector cells (*P* < 0.01). In addition, β_2_M shRNA-expressing ARP-1 cells secreted significantly less soluble β_2_M whereas β_2_M ORF-expressing ARP-1 cells secreted more compared with control cells (Supplementary Figure S2E; *P* < 0.01). Flow cytometry analysis showed a 70% reduction inβ_2_M shRNA-ARP-1 cells whereas β_2_M ORF-ARP-1 cells had a 2-fold increase in surface expression of β_2_M (Supplementary Figure S2F) and HLA-ABC (Supplementary Figure S2G) compared with control cells (*P* < 0.01).

**Figure 3:**
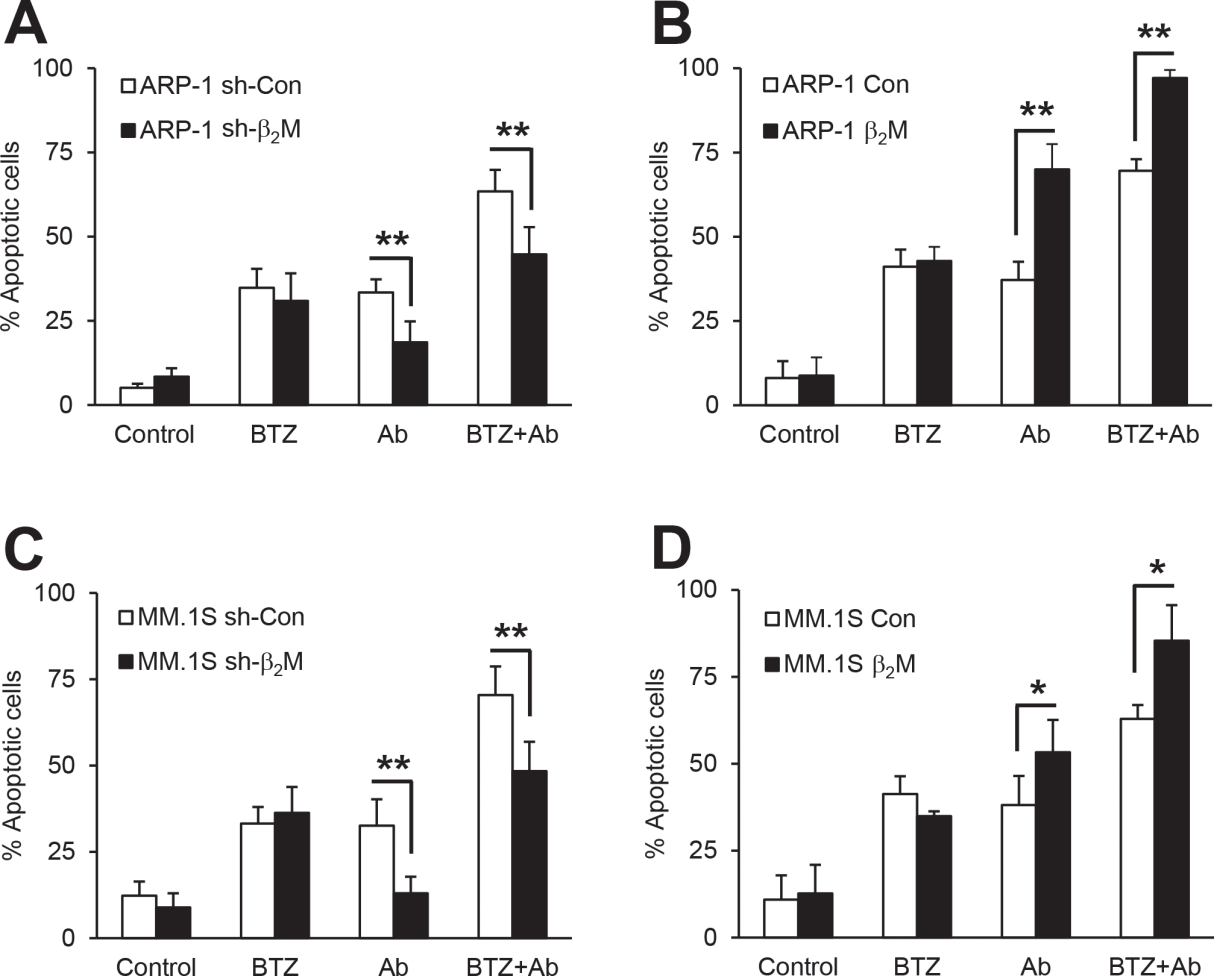
The efficacy of anti-β_2_M mAbs and BTZ combination treatment in β_2_M-knockdown and β_2_M-overexpression MM cells Non-specific (sh-con) or β_2_M shRNA (sh-β_2_M)-expressing, and stable control vector (con) or human β_2_M cDNA (β_2_M)-expressing ARP-1 **(A** and **B)** and MM.1S **(C** and **D)** cells were cultured in medium with or without addition of BTZ (5 nM) or anti-β_2_M mAbs (10 μg/mL), singly or in combination for 24 hours. Apoptosis was reduced in β_2_M shRNA-expressing ARP-1 **(A)** and MM.1S **(C)** cells receiving combination treatment compared with cells treated with BTZ only. Apoptosis was enhanced in β_2_M-overexpressing ARP-1 **(B)** and MM.1S **(D)** cells receiving combination treatment, compared with cells treated with BTZ only. Summarized data from three independent experiments are shown. *P < 0.05, **P < 0.01.

Next, the effects of anti-β_2_M mAb or BTZ treatment, singly or in combination, on MM cell apoptosis were examined in β_2_M-knockdown and β_2_M-overexpressing MM cells. After 24-hour treatment, anti-β_2_M mAb treatment alone induced apoptosis of control cells and enhanced apoptosis ofβ_2_M-overexpressing cells, but reduced apoptosis in β_2_M-knockdown cells; BTZ treatment alone induced apoptosis in all tested cells (Figure 3). Combination treatment did not enhance apoptosis in β_2_M-knockdown cells (Figure 3A and 3C) but did in β_2_M-overexpressing cells (Figure 3B and 3D), as compared with BTZ treated-only cells (*P* < 0.01). These results indicate that the enhanced effects of combination treatment depend on MM cell β_2_M expression.

### Combination of anti-β_2_M mAbs and BTZ reduces BTZ-induced autophagy

**Figure 4:**
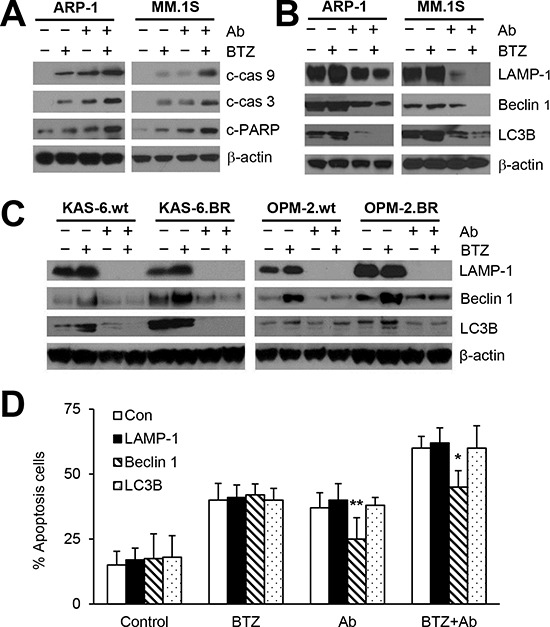
Anti-β_2_M mAbs and BTZ combination treatment reduces BTZ-induced autophagy activation MM cells were cultured in medium with or without addition of BTZ (5 nM) or anti-β_2_M mAbs (10 μg/mL), singly or in combination for 24 hours. Representative images of Western blot analysis **(A)** showing the levels of cleaved caspase 9 (c-cas9), caspase 3 (c-cas3), and PARP (c-PARP) in ARP-1 and MM.1S cells. Representative images of Western blot analysis showing the levels of the autophagy proteins LAMP-1, Beclin-1, and LC3B in **(B)** ARP-1 and MM.1S cells and **(C)** KAS-6.wt, KAS-6.BR, OPM-2.wt, and OPM-2.BR cells. **(D)** Annexin-V binding assay showing that rescuing Beclin 1 but not LC3B or LAMP-1 reduced apoptosis in ARP-1 cells treated with anti-β_2_M mAbs alone or with mAbs plus BTZ. The experiments were carried out in triplicate. β-actin served as protein loading control. *P < 0.05, **P < 0.01.

To further determine the enhanced effects of combination treatment on MM cell apoptosis, we evaluated caspase cascades in MM cells treated for 24 hours. In ARP-1 and MM.1S cells, BTZ or anti-β_2_M mAb treatment alone resulted in an accumulation of cleaved caspase 9, caspase 3, and PARP, and the combination treatment enhanced the caspase cleavage (Figure 4A). These findings were in line with the annexin-V binding assay results (Figure 1) and suggest that anti-β_2_M mAbs plus BTZ enhances caspase activation in MM cells.

Cell death and survival are regulated by the crosstalk between apoptosis and autophagy [32]. Recent studies have shown that autophagy activation plays a role in BTZ drug resistance in patients with cancer [11]. We therefore determined the effects of 24-hour anti-β_2_M mAb and BTZ combination treatment on autophagy activation. Treatment with BTZ alone up-regulated the expression of autophagy proteins LAMP-1, Beclin 1, and LC3B, whereas treatment with anti-β_2_M mAbs alone or in combination with BTZ decreased expression in ARP-1 and MM.1S cells (Figure 4B). Next, the effects of anti-β_2_M mAb or BTZ treatment, singly or in combination for 24 hours, on the expression of autophagy proteins in KAS-6.wt, KAS-6.BR, OPM2.wt, and OPM2.BR cells were examined. As shown in Figure 4C, autophagy protein expression was higher in BTZ-resistant cell lines compared with BTZ-sensitive cell lines. BTZ treatment alone up-regulated the expression of LAMP-1, Beclin 1, and LC3B whereas anti-β_2_M mAb treatment alone or combined with BZT down-regulated the expression in both BTZ-resistant and -sensitive cell lines. These results indicated that combining anti-β_2_M mAbs and BTZ overcomes BTZ-induced autophagy in both BTZ-resistant and -sensitive MM cells.

To determine which autophagy proteins mediate BTZ resistance, we rescued LAMP-1, Beclin 1, and LC3B expression in ARP-1 cells by infection with lentivirus containing human LAMP1, Beclin-1, or LC3B ORFs, respectively. After 24-hour treatment, rescuing Beclin 1, but not LC3B or LAMP-1, reduced apoptosis in ARP-1 cells treated with anti-β_2_M mAbs alone or in combination with BTZ (Figure 4D), indicating that Beclin 1 is responsible for the anti-β_2_M mAb-induced inhibition of autophagy.

### Combination treatment down-regulates autophagy by inhibiting BTZ-activated NF-κB p65 signaling

**Figure 5:**
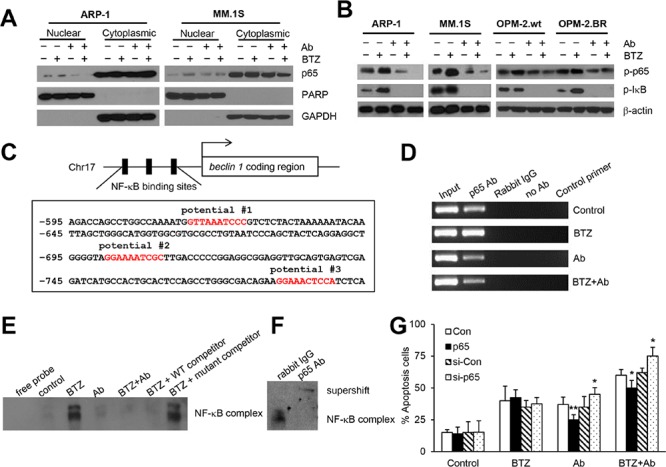
Anti-β_2_M mAbs and BTZ combination treatment down-regulates BTZ-induced NF-κB p65 activity After 24 hours of treatment with BTZ or anti-β_2_M mAbs, singly or in a combination, MM cells were harvested and the cytoplasmic and nuclear proteins were extracted. **(A)** Representative images of Western blot analysis showing nuclear and cytoplasmic NF-κB p65 in ARP-1 and MM.1S cells. PARP and GAPDH served as nuclear and cytoplasmic loading controls, respectively. **(B)** Representative images of Western blot analysis showing phosphorylated NF-κB p65 and phosphorylated IκB-α in ARP-1, MM.1S, OPM-2wt, and OPM-2.BR cells. β-actin served as a protein loading control. **(C)** Schematic diagram of NF-κB binding sites in the *beclin 1* promoter region. The locations of three potential NF-κB binding sites are indicated. **(D)** Representative ChIP assay images showing the ability of NF-κB p65 to bind to the *beclin 1* promoter in BTZ-, mAb- or mAb plus BTZ-treated ARP-1 cells. Chromatin was extracted from the treated cells, and the DNA was precipitated with p65 antibody, then analyzed by qPCR. The input served as an internal control, and samples treated with rabbit IgG, or no antibody, or control primer of non-transcribed region served as negative controls. Nuclear proteins were extracted from ARP-1 cells after 24-hour treatment for EMSA assay. **(E)** Binding of potential #2 probe was presented after BTZ treatment and reduced in the mAb- or combination treatment. Cold competition was performed using unlabeled probes as indicated. **(F)** Supershift assay performed with p65 antibody to investigate the BTZ-induced p65 binding complex to *beclin 1* promoter. Rabbit IgG served as a negative control. **(G)** Rescuing or knocking down p65 in ARP-1 cells showing reduced or enhanced apoptosis by annexin-V binding assay. All experiments were carried out in triplicate. *P < 0.05, **P < 0.01.

Constitutive NF-κB activity in cancer cells can induce BTZ resistance [33, 34]. Therefore, we wondered whether anti-β_2_M mAbs inhibited BTZ-induced autophagy by inhibiting NF-κB signaling. After 24-hour treatment, cytoplasmic and nuclear protein fractions of ARP-1 and MM.1S cells were extracted and detected by Western blotting. As shown in Figure 5A, BTZ treatment alone induced the translocation of NF-κB p65 into nuclei, whereas anti-β_2_M mAb treatment alone or in combination with BTZ had no such effect. In addition, BTZ treatment alone increased the phosphorylation levels of p65 and IκB-α in ARP-1, MM.1S, OPM-2.wt, and OPM-2.BR cells after 24-hour treatment, whereas anti-β_2_M mAb treatment alone or in combination with BTZ reduced the levels of phosphorylated p65 and IκB-α (Figure 5B). These results indicate that BTZ alone activates NF-κB signaling, whereas anti-β_2_M mAb and BTZ combination treatment reduces the signaling.

### Combination treatment inhibits BTZ-induced NF-κB p65 binding to the *beclin 1* promoter

Sequence analysis of the *beclin 1* promoter region showed 3 putative NF-κB binding sites from –615 to –789 bp (Figure 5C). Chromatin immunoprecipitation (ChIP) assay verified that 24-hour treatment with BTZ treatment alone up-regulated p65 binding to the *beclin 1* promoter in ARP-1 cells, but anti-β_2_M mAb treatment alone or in combination with BTZ reduced the binding (Figure 5D). To further confirm that BTZ induced p65 binding to the *beclin 1* promoter, electrophoretic mobility shift assay (EMSA) was performed. As shown in Supplementary Figure S3, after 24-hour treatment, potential #2 probe could bind to ARP-1 nuclear proteins, but potential #1 and potential #3 probes could not. Further analysis of the potential #2 probe confirmed an intense band in the BTZ treatment group, but only faint bands in the anti-β_2_M mAb and combination treatment groups (Figure 5E and Supplementary Figure S3), indicating that BTZ treatment enhanced protein binding to the *beclin 1* promoter but anti-β_2_M mAbs or combination treatment inhibited the binding. For DNA competition experiments, the inducible band could be shifted off completely by unlabeled potential #2 probe, but not by unlabeled mutant potential #2 probe (Figure 5E). After BTZ treatment, p65 antibody supershifted the identified band, indicating that BTZ presence leads to p65 binding of the *beclin 1* promoter (Figure 5F). These results indicate that anti-β_2_M mAbs reduce BTZ-induced autophagy by inhibiting NF-κB p65 binding to the *beclin 1* promoter.

In addition, rescuing p65 by lentiviral infection of human p65 ORF treated with anti-β_2_M mAbs alone or combined with BTZ for 24 hours reduced apoptosis, whereas knocking down p65 by lentiviral infection with human p65 shRNA increased apoptosis of ARP-1 cells (Figure 5G). Taken together, these results indicate that anti-β_2_M mAbs enhanced BTZ treatment efficacy via an NF-κB–Beclin 1 signaling pathway.

### Anti-β_2_M mAbs enhance the anti-MM effects of BTZ *in vivo*

**Figure 6:**
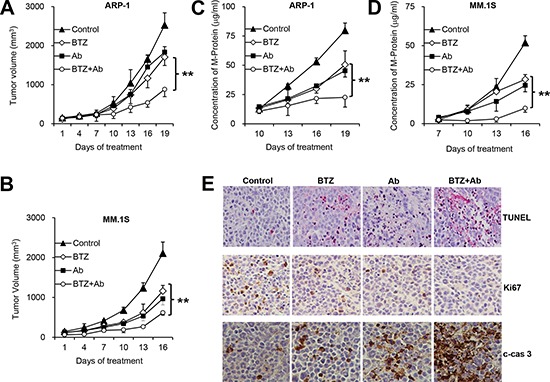
Anti-β_2_M mAbs enhance anti-MM effects of BTZ *in vivo* Shown are tumor volumes **(A, B)** and M-protein levels **(C, D)** in ARP-1 or MM.1S tumor-bearing mice, respectively (*n* = 4), treated with mouse IgG1 or DMSO (control), BTZ, anti-β_2_M mAbs (Ab), or the combination of BTZ and anti-β_2_M mAbs (BTZ+Ab). ARP-1 or MM.1S cells were subcutaneously injected into SCID mice. At 3 to 4 weeks after MM cell injection, mice were intraperitoneally injected with BTZ (0.1 mg/kg) or subcutaneously around tumors with anti-β_2_M mAbs (0.6 mg/kg), singly or in combination, every 3 days for 3 weeks. Tumor volumes were measured every 3 days after treatment. The level of circulating human kappa or lambda chain in mouse serum was measured by ELISA. **(E)** Representative images of *in situ* TUNEL assay and immunohistochemistry of Ki67 and cleaved caspase 3 (c-cas 3) showing MM tumor cell apoptosis and proliferation. **P < 0.01.

We examined the therapeutic effects of anti-β_2_M mAb and BTZ combination treatment *in vivo* in a xenograft MM SCID mouse model. To detect the effects of combination treatment, low and nontherapeutic doses of BTZ and anti-β_2_M mAbs were chosen based on our previous studies [25, 35]. Although treatment with anti-β_2_M mAbs or BTZ reduced tumor volume (*P* < 0.05, versus control mice), combination treatment was more efficacious than BTZ alone (Figure 6A and 6B; *P* < 0.01). Tumor burden was further assessed by measuring serum M-protein levels by ELISA (Figure 6C and 6D; *P* < 0.01). No change in body weight was found in treated groups (data not shown), suggesting that the combination treatment probably had no toxic effect.

Greater numbers of apoptotic tumor cells were detected by TdT-mediated dUTP nick-end labeling (TUNEL) assay in ARP-1 tumor-bearing mice treated with BTZ or anti-β_2_M mAbs, compared with mice treated with DMSO or mouse IgG1. Anti-β_2_M mAb and BTZ combination treatment showed additive effects on induction of MM cell apoptosis compared with treatment with BTZ alone (Figure 6E). Immunohistochemistry in ARP-1 tumor-bearing mice revealed that cells positive for Ki67 decreased after combination treatment, and cells positive for cleaved caspase 3 increased after combination treatment, compared with BTZ treatment alone (Figure 6E). These data indicate that combination of anti-β_2_M mAbs and BTZ enhances BTZ's therapeutic effects against MM *in vivo*.

## DISCUSSION

Chemotherapy is the most effective treatment for MM currently. Several new drugs have been developed to prolong patient survival [36, 37]. However, the application of these drugs, such as BTZ, usually induces drug resistance, and quick relapse is common [38, 39]. mAbs are emerging as a major new treatment that confers great benefits [40]. In this study, we determined that the combination of anti-β_2_M mAbs and BTZ was more effective against MM than either agent alone. More importantly, we found that anti-β_2_M mAbs overcome BTZ resistance by inhibiting BTZ-induced autophagy.

Anti-β_2_M mAbs enhanced the anti-MM effects of BTZ in a panel of established human MM cell lines and primary MM cells from patients. These findings indicate the potential of anti-β_2_M mAbs and BTZ combination treatment as a therapeutic strategy against MM. Moreover, anti-β_2_M mAbs re-sensitized BTZ-resistant MM cells to BTZ treatment, and the enhanced effects of the combination treatment correlated with the expression of surface β_2_M on MM cells. Therefore, combination treatment with anti-β_2_M mAbs and BTZ has the potential to impact a larger and heterogeneous patient population with β_2_M-expression, and to be effective in patients with relapse or who develop tumors resistant to conventional treatment with BTZ.

Mechanistic studies showed that the combination of anti-β_2_M mAbs and BTZ resulted in an accumulation of cleaved caspase 9, caspase 3 and PARP, and inhibition of the autophagy proteins LAMP-1, Beclin 1, and LC3B. Treatment with a low concentration of BTZ had only a minor effect on caspase cleavage but could induce autophagy. Notably, we found that anti-β_2_M mAbs inhibited BTZ-induced autophagy in a dose-dependent manner (data not shown). Thus, the combination of anti-β_2_M mAbs and BTZ may provide an approach to overcome BTZ drug resistance through the inhibition of BTZ-induced autophagy.

Recent research demonstrated that BTZ can induce canonical NF-κB activation by down-regulating constitutive IκB-α expression in MM cells [41]. Other studies found that BTZ treatment of primary effusion lymphoma cells failed to inhibit NF-κB activation [42]. In line with these reports, our data showed that BTZ activated NF-κB transcription activity by increasing NF-κB p65 nuclear translocation and p65 phosphorylation in MM cells. The combination of anti-β_2_M mAbs and BTZ significantly reduced both the NF-κB p65 nuclear translocation and p65 phosphorylation. Other groups have reported novel NF-κB p65 consensus sites in the *beclin 1* promoter and demonstrated that NF-κB p65 positively modulated canonical autophagy in various human tumor cell lines [43]. Our ChIP and EMSA assays verified that anti-β_2_M mAb treatment inhibited BTZ-induced NF-κB p65 binding to the *beclin 1* promoter.

The enhanced anti-MM effect of the combination therapy was also found *in vivo*. Combination treatment with anti-β_2_M mAbs and BTZ inhibited tumor growth and serum M-protein level compared with either agent alone. These results underscore a potential clinical development strategy by combining anti-β_2_M mAbs and BTZ to treat MM patients, which could lower the doses of BTZ and anti-β_2_M mAbs needed while enhancing their anti-tumor effects, and more importantly, reduce BTZ- and anti-β_2_M mAb-induced toxicity.

As shown in our schematic diagram of signaling pathways (Supplementary Figure S4), BTZ induces caspase cleavage and apoptosis of MM cells, resulting in drug sensitivity to BTZ treatment. However, BTZ could also enhance *beclin 1* transcription by increasing NF-κB p65 binding to the *beclin 1* promoter, leading to autophagy activation. Activated autophagy inhibits MM cell apoptosis and promotes cell survival, resulting in drug resistance to BTZ treatment (Supplementary Figure S4A). When BTZ is combined with anti-β_2_M mAbs, NF-κB p65 transcription activities and BTZ-induced autophagy are inhibited, while caspase cleavage and MM cell apoptosis are increased, resulting in re-sensitization of MM cells to BTZ treatment (Supplementary Figure S4B). Thus, our study strongly suggests that anti-β_2_M mAbs can overcome BTZ drug resistance in patients.

In conclusion, we for the first time demonstrate that anti-β_2_M mAbs prevent BTZ drug resistance and enhance BTZ anti-MM efficacy by reducing autophagy protein expression via NF-κB signaling, which provides a rationale for combining these drugs to improve patient outcomes in MM. Thus, our studies provide new insight for the clinical development of anti-β_2_M mAbs to overcome chemotherapy drug resistance and improve MM patient survival.

## MATERIALS AND METHODS

### Reagents, MM cell lines, and primary MM cells

Anti-β_2_M mAbs (clone D1) were generated as previously described [25]. Mouse IgG1 (BioLegend) was used as an isotype control. BTZ (PS-341; Millennium) was dissolved in DMSO at 10 mM as a stock solution. Human ARP-1 and ARK cells were established at the University of Arkansas for Medical Sciences from BM aspirates of patients with MM [44], MM.1S was kindly provided by Dr. Steven Rosen of Northwestern University (Chicago, IL), and U266 cells were purchased from the American Type Culture Collection (ATCC). The BTZ-sensitive (wild-type, wt) and BTZ-resistant (BR) MM cell lines KAS-6.wt, KAS-6.BR, OPM-2.wt, and OPM-2.BR were generated by Dr. Robert Z. Orlowski as previously described [31]. Primary CD138^+^ MM cells were isolated from BM aspirates of MM patients according to approved IRB protocols of The University of Texas MD Anderson Cancer Center and Cleveland Clinic. All cells were cultured in RPMI-1640 medium supplemented with 10% fetal bovine serum and maintained at 37°C with 5% CO_2_.

### Lentiviral infection of MM cells with shRNA and ORF expression clone transfection

MM cells were infected with lentivirus containing human β_2_M or p65 shRNAs or lentivirus containing human β_2_M, LAMP1, Beclin-1, LC3B, or p65 ORFs (Genecopoeia) according to the manufacturer's protocol to knockdown or overexpress specific gene, respectively. Non-specific shRNA or control vector was used as controls. Stable cell line screening was performed with 800 μg/mL neomycin (Sigma) for 4 weeks, and positive cells were selected.

### Extraction of cytoplasmic and nuclear proteins

MM cell cytoplasmic and nuclear protein fractions were extracted using NE-PER extraction reagents according to the manufacturer's protocol (Pierce Biotechnology). Nuclear protein extracts were used for EMSA. Cytoplasmic and nuclear protein extracts were used for detecting NF-κB p65 by Western blotting. PARP or GAPDH served as a nuclear or cytoplasmic internal control, respectively.

### Western blotting

Western blotting was conducted as previously described [25]. Mouse anti-β_2_M mAbs (Santa Cruz Biotech) were used to detect β_2_M protein. Rabbit polyclonal antibodies against cleaved caspase 9, cleaved caspase 3, cleaved PARP, LAMP-1, Beclin 1, LC3B, non-phosphorylated p65, phosphorylated p65, phosphorylated IκB-α, GAPDH, PARP, and β-actin were obtained from Cell Signaling Technology.

### qPCR

Total RNA was isolated using an RNeasy kit (Qiagen). Total RNA (1 μg) was reverse transcribed using a SuperScript II (Invitrogen) reverse transcriptase PCR kit; 1 μL of the final cDNA was used for qPCR amplification with SYBRGreen using a StepOnePlus real-time PCR system (Applied Biosciences). The primers for amplification were: β_2_M-F 5′-AAT TGA AAA AGT GGA GCA TTC AGA-3′; β_2_M-R 5′-GGC TGT GAC AAA GTC ACA TGG TT-3′; GAPDH-F 5′-CAC TCC TCC ACC TTT GAC G-3′; and GAPDH-R 5′-ACC ACC CTG TTG CTG TAG C-3′. Gene expression levels were normalized to *GAPDH* levels.

### Analysis of surface β_2_M and HLA-ABC and cell apoptosis by flow cytometry

APC-conjugated mAbs against human β_2_M, HLA-ABC, and isotype control were obtained from BioLegend. An apoptosis assay was performed as previously described [25]. FITC-labeled annexin-V antibody and propidium iodide (PI) were purchased from Life Technologies. Data were acquired with a flow cytometer (FACSCalibur; BD Biosciences).

### ELISA

Cell culture supernatants were collected, and secreted β_2_M was quantified with a human β_2_M Quantikine IVD ELISA Kit (R&D Systems). Serum M-protein levels were measured in SCID mice injected with ARP-1 by using the Human Kappa ELISA Kit or MM.1S cells using the Human Lambda ELISA Kit (Bethyl Laboratories).

### ChIP assay

ChIP assay was performed with a ChIP assay kit (Millipore) according to the manufacturer's instructions. Chromatin was extracted from ARP-1 cells. Anti-NF-κB p65 antibody and isotype control (Cell Signaling Technology) were used for the chromatin immunoprecipitation. The precipitated DNA was analyzed by qPCR with the following primer sets for the region surrounding the NF-κB binding sites at the *beclin 1* promoter: F 5′-AGA CCA GCC TGG CCA AAA TGG T-3′ and R 5′-TGA GAT GGA GTT TCC TTC TGT CG-3′. Values were subtracted from control IgG values and normalized to corresponding input control.

### EMSA

Probes were labeled at the 3′ end with biotin (Biotin 3′ End DNA Labeling Kit; Pierce Biotechnology), following the manufacturer's instructions. Oligonucleotides used as probe or competitor were synthesized as: potential #1, 5′-AAA TGG TTA AAT CCC GTC TCT A-3′; potential #2, 5′-GGG TAG GAA AAT CGC TTG ACC C-3′; potential #2 mutant, 5′-GGG TAG CAA AAT CCC TTG ACC C-3′; potential #3, 5′-GAC AGA AGG AAA CTC CAT CTC A-3′. EMSA was performed using the LightShift Chemiluminescent EMSA Kit (Pierce Biotechnology). The DNA-protein complexes were separated on 6% native polyacrylamide gels in 0.5% Tris-borate buffer. For DNA competition experiments, a 100-fold molar excess of unlabeled oligonucleotide was added before incubation. For supershift experiments, 2 μL of anti-NF-′B p65 antibody or isotype control was added to the completed binding reaction mixture [45].

### *In vivo* tumor xenograft mouse models

Six-week-old male SCID mice (Jackson Laboratory) were injected subcutaneously in the right flank with 1 × 10^6^ ARP-1 or MM.1S cells. At 3 to 4 weeks later when palpable tumors (5 mm in diameter) developed, mice (*n* = 4 per group) were intraperitoneally injected with BTZ (0.1 mg/kg), subcutaneously injected around tumors with anti-β_2_M mAbs (0.6 mg/kg), either singly or in combination every 3 days for 3 weeks. Control mice were injected with equal amounts of mouse IgG1 or DMSO. Tumors were measured every 3 days with calipers, and tumor volumes (mm^3^) were calculated as (width^2^ × length)/2. Mice were humanely sacrificed when moribund or when subcutaneous tumors reached 15 mm in diameter. All mice were maintained in facilities accredited by the American Association of Laboratory Animal Care, and the studies were approved by the Institutional Animal Care and Use Committees of The University of Texas MD Anderson Cancer Center and Cleveland Clinic.

### *In situ* apoptosis assay and immunohistochemistry

*In situ* tumor cell apoptosis was determined by using a TUNEL assay kit (Boehringer–Mannheim). Immunohistochemistry was conducted as previously described [46]. Ki67 and cleaved caspase 3 expression were detected using specific antibodies (Cell Signaling Technology). Sectioned tumor tissue was embedded in paraffin. Three slides from each treatment group were evaluated. Six fields were arbitrarily selected for examination, using a defined rectangular field area at × 200 magnification, and positive staining cells were counted in each field.

### Statistical analysis

The Student's *t*-test was used to compare various experimental groups. A *P* value < 0.05 was considered statistically significant. Unless otherwise indicated, the values provided are means and standard deviations (SD).

## SUPPLEMENTARY FIGURES


