# Supplementary material for: Anti-β2-microglobulin monoclonal antibodies overcome bortezomib resistance in multiple myeloma by inhibiting autophagy
Source: Oncotarget. 2015 Mar 31;6(11):8567–78. doi: 10.18632/oncotarget.3251 (PMC4496167; doi:10.18632/oncotarget.3251)
Supplement: Supplementary file 1 [file oncotarget-06-8567-s001.pdf]

SUPPLEMENTARY FIGURES

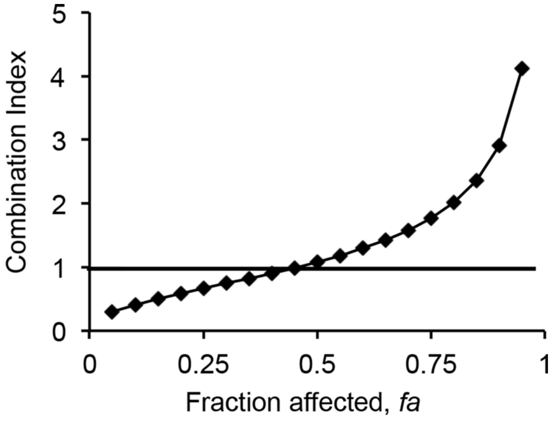

Supplementary Figure S1: Combination Index (CI) isobol equation to study drug interactions between BTZ and anti-β2M mAbs. The CI theory of Chou-Talalay offers quantitative definitions for additive effect (CI = 1), synergism (CI < 1), and antagonism (CI > 1) in determining the effect of drug combinations. The combination of BTZ and anti-β_2M mAbs has a synergistic effect (CI < 1) when combined at low concentrations.

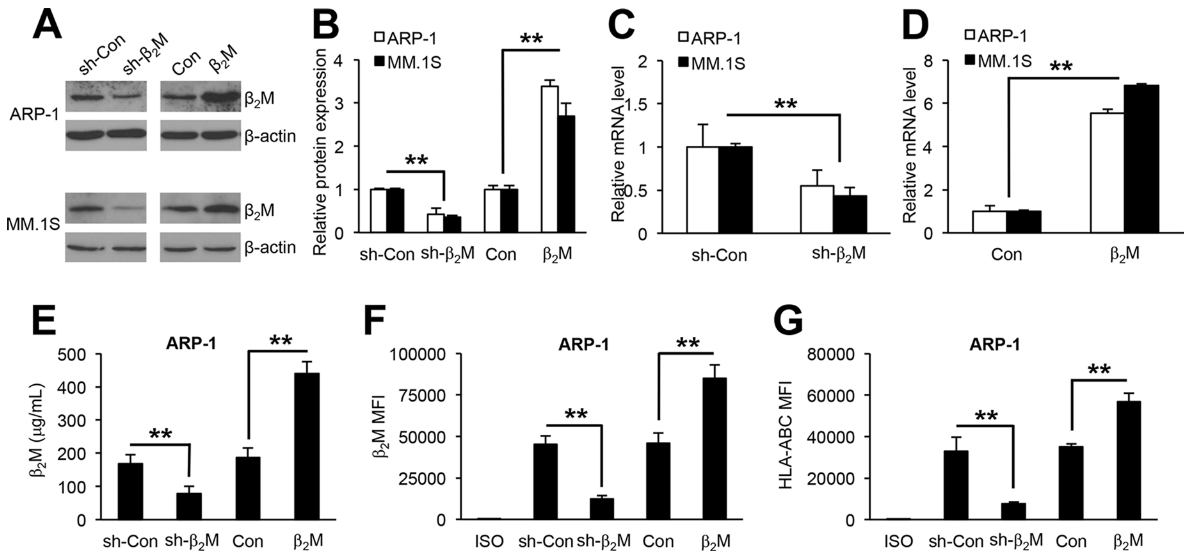

Supplementary Figure S2: Establishment of β2M knockdown and overexpression of stable ARP-1 and MM.1S cell lines. Representative images **(A)** and quantification **(B)** of Western blot analysis showing the protein levels of β_2M in stable non-specific (sh-con) or β_2M shRNA (sh-β_2M)-expressing, and in stable control vector (con) or human β_2M cDNA (β_2M)-expressing ARP-1 and MM.1S cells. β-actin served as protein loading control. qPCR showing the relative mRNA levels of the *β_2M* gene in the β_2M knockdown **(C)** and overexpression **(D)** ARP-1 and MM.1S cells. *GAPDH* mRNA served as an internal control. **(E)** Secreted β_2M level in medium from cultured β_2M knockdown or overexpressionARP-1 cells, as detected by ELISA. Also shown are the mean fluorescence intensity (MFI) for the cell surface expression of β_2M **(F)** and HLA-ABC **(G)** on β_2M-knockdown or β_2M-overexpressing ARP-1 cells, as detected by flow cytometry. Isotype antibodies (ISO) served as controls. Summarized data from three independent experiments are shown. $^{**}P < 0.01$.

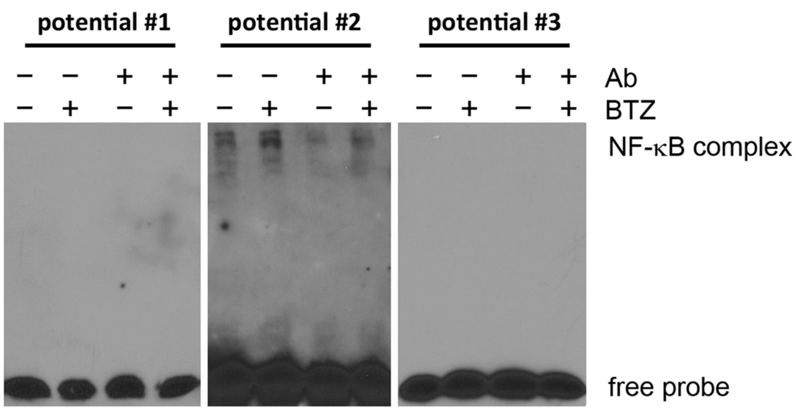

Supplementary Figure S3: Gel shift activities induced by combination treatment with three potential NF-κB binding sites on *beclin 1* promoter. Potential #2 probe bound nuclear proteins of ARP-1 cellnuclear extracts after 24-h treatment with BTZ (5 nM) or anti-β_2M mAbs (10 μg/mL), singly or in combination, whereas binding was not present with potential #1 and potential #3 probes.

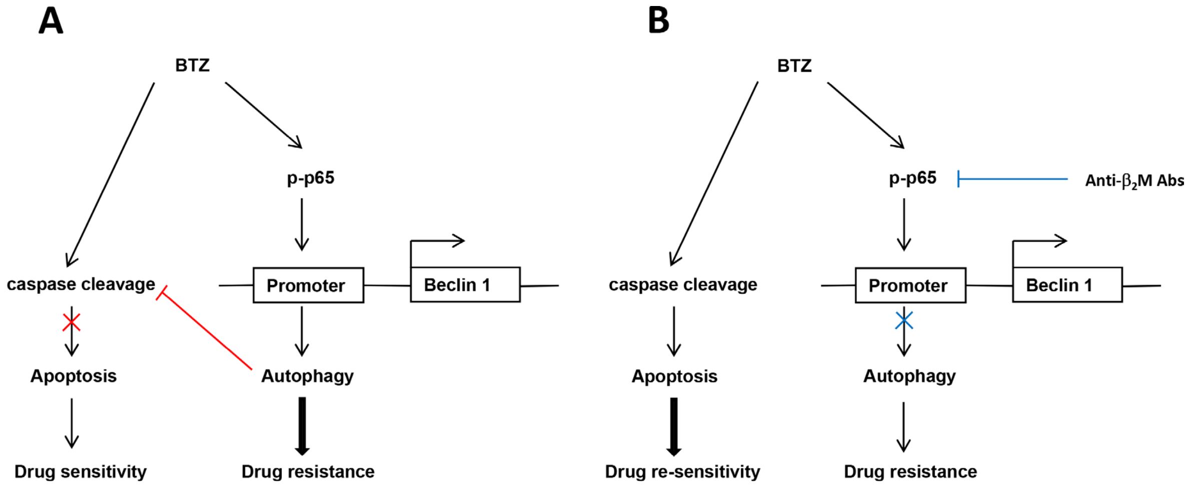

Supplementary Figure S4: Schematic diagram of the mechanism proposed to underly the ability of anti-β2M mAb to overcome MM drug resistance against BTZ. (A) Proposed mechanism by which BTZ increases caspase cleavage and induces MM cell apoptosis to result in MM cell drug sensitivity to BTZ. However, BTZ could also enhance *beclin 1* transcription via phosphorylating NF-κB p65, activating autophagy, and inhibiting caspase cleavage and cell apoptosis, resulting in drug resistance to BTZ. **(B)** The mechanism by which anti-β_2M mAbs and BTZ combination treatment inhibits BTZ-induced phosphorylation of NF-κB p65 and autophagy, and increases caspase cleavage and cell apoptosis to resensitize cells to BTZ.
